# Genome-wide DNA methylation detection by MethylCap-seq and Infinium HumanMethylation450 BeadChips: an independent large-scale comparison

**DOI:** 10.1038/srep15375

**Published:** 2015-10-20

**Authors:** Tim De Meyer, Pierre Bady, Geert Trooskens, Sebastian Kurscheid, Jocelyne Bloch, Johan M. Kros, Johannes A. Hainfellner, Roger Stupp, Mauro Delorenzi, Monika E. Hegi, Wim Van Criekinge

**Affiliations:** 1 Dept. of Mathematical Modelling, Statistics and Bioinformatics, Faculty of Bioscience Engineering, Ghent University, Ghent, Belgium; 2 Lab. of Brain Tumor Biology and Genetics, Department of Clinical Neurosciences, University Hospital Lausanne, Lausanne, Switzerland; 3 Swiss Institute of Bioinformatics, Lausanne, Switzerland; 4 Département de Formation et Recherche, University Hospital/University of Lausanne, Lausanne, Switzerland; 5 Neurosurgery, Department of Clinical Neurosciences, University Hospital Lausanne, Lausanne, Switzerland; 6 Division of Neuropathology, University Hospital Rotterdam, Rotterdam, The Netherlands; 7 Institute of Neurology, Medical University of Vienna, Vienna, Austria; 8 Department of Oncology, University Hospital Zurich, Zurich, Switzerland

## Abstract

Two cost-efficient genome-scale methodologies to assess DNA-methylation are MethylCap-seq and Illumina’s Infinium HumanMethylation450 BeadChips (HM450). Objective information regarding the best-suited methodology for a specific research question is scant. Therefore, we performed a large-scale evaluation on a set of 70 brain tissue samples, *i.e.* 65 glioblastoma and 5 non-tumoral tissues. As MethylCap-seq coverages were limited, we focused on the inherent capacity of the methodology to detect methylated loci rather than a quantitative analysis. MethylCap-seq and HM450 data were dichotomized and performances were compared using a gold standard free Bayesian modelling procedure. While conditional specificity was adequate for both approaches, conditional sensitivity was systematically higher for HM450. In addition, genome-wide characteristics were compared, revealing that HM450 probes identified substantially fewer regions compared to MethylCap-seq. Although results indicated that the latter method can detect more potentially relevant DNA-methylation, this did not translate into the discovery of more differentially methylated loci between tumours and controls compared to HM450. Our results therefore indicate that both methodologies are complementary, with a higher sensitivity for HM450 and a far larger genome-wide coverage for MethylCap-seq, but also that a more comprehensive character does not automatically imply more significant results in biomarker studies.

DNA-methylation is an epigenetic feature that is essential for a variety of biological processes, including imprinting, X-chromosome inactivation, differentiation and development. In humans, the methylation of cytosines particularly occurs at CpG-dinucleotides, which are often grouped in so-called CpG-islands, and is catalyzed by DNA methyltransferases. Gene promoter hypermethylation is typically associated with gene silencing, but recent research is demonstrating a more complex role with additional functions of non-promoter methylation in e.g. splicing, and relevant methylation of regions surrounding CpG-islands, namely CpG-island shores and shelves^1^.

Per definition, epigenetic marks can be mitotically inherited, which is crucial to maintain a stable expression signature. On the other hand, deviant DNA-methylation patterns have been demonstrated in a plethora of diseases, with cancer as particular example^2^,^3^,^4^,^5^. The high-throughput assessment of DNA-methylation across the genome is therefore not only crucial to deepen our insight in the regulation of biological processes as for instance gene expression, differentiation and development, but also to identify pathology associated aberrations, which might have potential clinical applications.

State-of-the-art methods to detect DNA-methylation on a genome-scale can be roughly divided into bisulfite and methylation enrichment based methods. Upon bisulfite treatment, unmethylated cytosines are deaminated into uracils, with subsequent basepairing behavior of thymines, whereas methylated cytosines remain intact^3^,^6^. The introduced sequence differences can be exploited using e.g. whole genome sequencing of bisulfite modified DNA, allowing a straightforward methylation quantification for each cytosine. Whole genome bisulfite sequencing is however cost-inefficient^7^, particularly in multi-sample studies. As a solution, only targeted regions of the genome are assessed with methods such as reduced representation bisulfite sequencing (RRBS)^8^ and targeted bisulfite sequencing^9^, or with the use of arrays.

The latter solution has been implemented in Illumina’s Infinium HumanMethylation BeadChips, which rely on the quantitative measurement of bisulfite induced (*i.e.* methylation status dependent) single nucleotide polymorphisms. The most recent generation BeadChips, HumanMethylation450, which we will further refer to as “HM450”, consist of two assay types, i.e. types 1 and 2. The HM450 type 1 assay employs two probes per CpG locus: one “methylated” and one “unmethylated” query probe located on corresponding “methylated” and “unmethylated” beads, respectively. After hybridization with the matching probe, single base extension incorporating a labeled nucleotide at the 3′ probe terminus will provoke either the “methylated” (M) or “unmethylated” (U) signal (same color channel, but on different beads). HM450 type 2 assays employ a single degenerate probe type that hybridizes to both variants, but different colors/channels are used to quantify and differentiate either methylated and unmethylated alleles^10^. Type 2 assays assess a far larger part of the genome, but it has been reported that the data distributions of the estimated methylation degrees differ between both assay types and that HM450 type 2 assay probe results are generally inferior^11^,^12^,^13^,^14^.

For both types of chemistries, methylation degrees are typically calculated as β-values, i.e. β = M/(U + M + α), with α a constant offset value (typically 100)^10^,^13^. Additionally, detection P-values are generated for each locus, indicating whether the result is reliable^10^. The combination of the rather straightforward data-analysis, good performance and low cost, has made Illumina’s HM450 (one of) the most popular tool(s) to assess DNA-methylation, although only in a human context and on a “genome-scale”, *i.e.* only a fraction of the full genome is assessed. An additional drawback is the fact that accurate probe design is complicated by the presence of SNPs, and that probes often lack specificity^15^. Though the raw data appear to be already highly reproducible^16^, additional preprocessing may further improve overall results and different widely used R Bioconductor packages are available for this purpose, e.g. *lumi*^17^ and *minfi*^18^.

An alternative cost-efficient, putatively genome-wide, option is to assess only methylated fragments, and is more broadly applicable than human-centric studies. Methylated DNA-fragments can be captured, after fragmentation, using either antibodies (methylated DNA immunoprecipitation, MeDIP)^19^ or Methyl-Binding Domains (MBD)^20^,^21^. Subsequent deep sequencing (MeDIP-seq or MBD-seq/MethylCap-seq) results in a genome-wide overview of each sample’s methylome. Here, the number of sequenced fragments for a specific locus is a proxy for its methylation degree. Although irrelevant for most studies - that typically compare the corresponding loci between cases and controls to identify differentially methylated regions - it should be noted that the number of fragments detected by these methods depends on the underlying CpG-density^22^.

This bias was observed in a recent study evaluating MeDIP-seq, MethylCap-seq, RRBS, and Infinium HumanMethylation27 BeadChips (“HM27”)^22^. This comparison also suggested that the MethylCap-seq methodology yielded the largest number of differentially methylated loci, whereas this was the lowest for the BeadChips. However, this study was based on a very limited sample set, and only considered the former HM27 which has a far more limited genomic coverage (~27,500 loci) than the currently widely used HM450 assay (~485,000 loci). HM450 has been compared with the cost-inefficient whole-genome bisulfite sequencing methodology, but only by the commercial developers of HM450 10. Therefore, we present an independent, large-scale comparison of HM450 with the previously best performing and affordable MethylCap-seq methodology, which allows further insights into the methodologies’ sensitivity and specificity and genome-wide character. For this comparison, we analyzed a total of 70 brain tissue samples, including 65 glioblastoma and 5 non-tumoral brain tissues.

## Results

Summary statistics for the HM450 and MethylCap-seq data for each of the 70 brain tissue samples (65 glioblastoma and 5-non tumoral samples) are available in Supplementary Table S1. Average beta-values were typically around 0.5 for HM450. Functional median sequencing depth (uniquely mapping fragments) was approximately 4 million (interquartile range 3.4–5.8 million), and ranged up to 16.5 million fragments. The average (standard deviation) MethylCap-seq coverage for the assessed HM450 loci was 0.60 (0.33). Since coverages were too low for a quantitative comparison, we particularly focused on the inherent capacity of each methodology to detect methylated loci. As explained in the methods section, for MethylCap-seq, loci were considered to be methylated when at least one captured fragment could be mapped on that specific position.

### MethylCap-seq - HM450 concordance depends on locus characteristics

**Figure 1:**
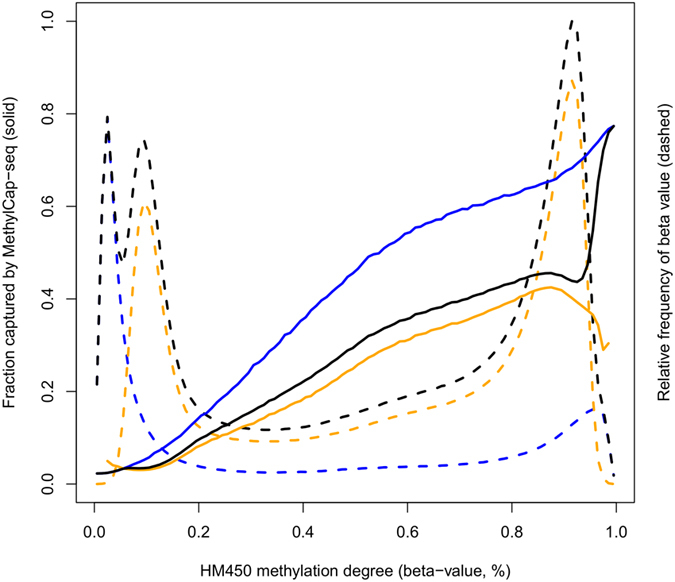
Concordance between MethylCap-seq and HM450 results. The fraction of fragments captured by MethylCap-seq (solid lines) are plotted as a function of HM450 methylation degrees (beta-values, binned per 1%), for both types of HM450 assays: type 1 (blue), type 2 (orange), and combined (black). The beta-value distributions are depicted as dashed lines for both types individually (type 1, blue; type 2, orange) and combined (black).

First, concordance between both methodologies was evaluated by plotting the binary MethylCap-seq data as a function of HM450 beta-values (Fig. 1). Over all samples, the beta-values were binned (100 equidistant bins) and for each bin the average binary MethylCap-seq value (= fraction that was captured by MethylCap-seq) was plotted. An overview of the beta-value distributions is also displayed in Fig. 1, depicting the fraction of beta-values present in each bin, for each assay type. An almost linear trend to increasing MethylCap-seq capturing for higher beta-values was observed, with virtually no capture for the lowest beta-values.

Over all samples, up to 80% of those loci indicated to be heavily methylated by HM450 type 1 assays (beta-value ≅ 1) were also picked up by MethylCap-seq, whereas this was as low as 40% for HM450 type 2 assays. This discrepancy between both HM450 assay types was also observed when considering the MethylCap-seq coverage values instead of the binary fractions (Supplementary Fig. S1). It can be hypothesized that the lower concordance for type 2 assay probes might be attributed to an inherent lower quality of these probes, in line with the already reported results^12^,^13^. On the other hand, type 2 assay probes have been designed to assess different genomic regions than type 1 probes, featuring different characteristics such as a significantly lower content of CpGs (median 4 vs. 7 for type 1, P < 2.2E-16). The lower concordance could therefore also be explained by the documented CpG^22^,^23^ and GC^23^,^24^ content biases of the MethylCap-sequencing methodology, providing an alternative hypothesis for our observation.

**Figure 2:**
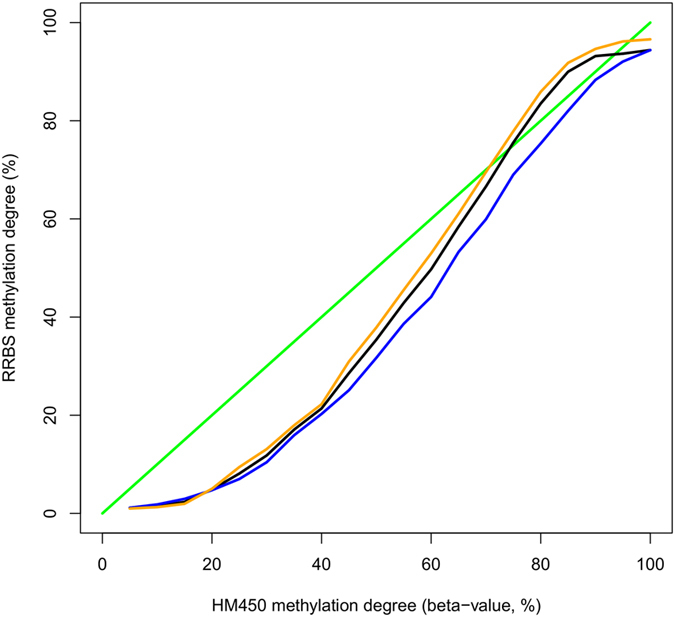
Concordance between RRBS and HM450 results. The average RRBS methylation degree is plotted as a function of methylation degrees (beta-values, binned per 5%) for both types of HM450 assays: type 1 (blue) and type 2 (orange), and combined (black). The green line reflects unbiased concordance between both methods (theoretical).

Therefore, we evaluated whether there was an effect of assay type on data quality by studying the concordance between HM450 and RRBS data generated by the ENCODE consortium^25^ for three cell lines (see Materials and Methods). Data were plotted in a similar fashion as visualized in Fig. 1, but raw HM450 methylation degrees were averaged for 5% beta-value bins. The comparison revealed that concordance was slightly *higher* for type 2 assay probes (Fig. 2), as was also observed for each individual cell line (data not shown). This indicates that the observed assay type effect can be attributed to MethylCap-seq specific biases, most likely related to the CpG- and GC-content of the broader probe target region, which might respectively affect the capturing and sequencing steps^22^,^23^,^24^.

Although overall correlation between HM450 and RRBS results was very good (Spearman correlation, type 1 assay: rho ~0.77, type 2 assay: rho ~0.89, all P < 2.2E-16), it is clear from Fig. 2 that HM450 beta-values also provide biased estimates of the actual methylation degrees, as probably more accurately reflected by the bisulfite sequencing data. Since both MethylCap-seq and HM450 methodologies have inherent strengths and weaknesses, neither can be considered as gold standard to assess sensitivity and specificity. Ideally, an independent gold standard methodology such as whole genome or reduced representation bisulfite sequencing should be used. However, beside the fact that bisulfite sequencing has its own limitations, the application on 70 samples for mere validation purposes is practically and financially not feasible. Therefore, a Bayesian modeling procedure^26^ was applied that avoids the selection of one methodology as reference. Bayesian estimation is known to be a valuable method to assess the unbiased conditional sensitivity and specificity without external standard^26^,^27^,^28^,^29^.

### Conditional sensitivity and specificity assessment of both methodologies

For the Bayesian analyses, also the HM450 data were dichotomized, using three different thresholds (beta-values of 0.2 to 0.4) for absence/presence of methylation. The two HM450 assay types were treated separately. A beta-value threshold of 0.1 was also evaluated but yielded discrepant results and appeared to be too low to define methylation status, in particular for type 2 assays (data not shown). Two samples with low MethylCap-seq sequencing depths were not considered here, as these would also be removed in normal experimental settings (<2 million mapped fragments). For each assay type and beta-value threshold presented, moderately to very good converging models were obtained for >90% of the remaining samples, indicating overall reliable parameter estimates. The lack of convergence of some models appeared to be more frequent for beta-value thresholds of 0.1 (data not shown) and 0.2 (Supplementary Fig. S2 and S3).

Results for sensitivity and specificity are presented for each assay type separately in Table 1. Differences mentioned *infra* are absolute differences (but mentioned as percentages as also specificity and sensitivity are indicated as percentages). Stated significant differences were so for each beta-value threshold at the 0.005 level (paired t-test, paired on sample). Overall, specificity and sensitivity results estimated by Bayesian models appear to be roughly similar for the different thresholds (maximal difference 5%). As expected, the estimated true prevalence of methylation, i.e. the number of methylated loci, decreased for increasing beta-value thresholds. Sensitivity and specificity appeared to be clearly dependent on methodology but also on assay type. For HM450, significantly higher sensitivities (~15%) and (slightly) lower specificities (~5%) were observed for type 2 assay loci. In line with results mentioned above, MethylCap-seq sensitivities were significantly lower for type 2 assay loci (~18%).

Overall, specificity was adequate for HM450 (>80%), but significantly higher for MethylCap-seq (>92%), implying a high ability to correctly classify unmethylated CpGs for both platforms. Whereas mean sensitivity values for HM450 were satisfying for type 1 assay loci (>70%) and good for type 2 assays (>87%), mean values for MethylCap-seq were significantly inferior: low for type 1 assays (>50%) and clearly poor for type 2 assays (32–36%). However, the major differences between minimum and maximum sensitivities for MethylCap-seq (Table 1) suggest that varying sequencing depths (cf. Supplementary Table S1) may have an impact.

**Figure 3:**
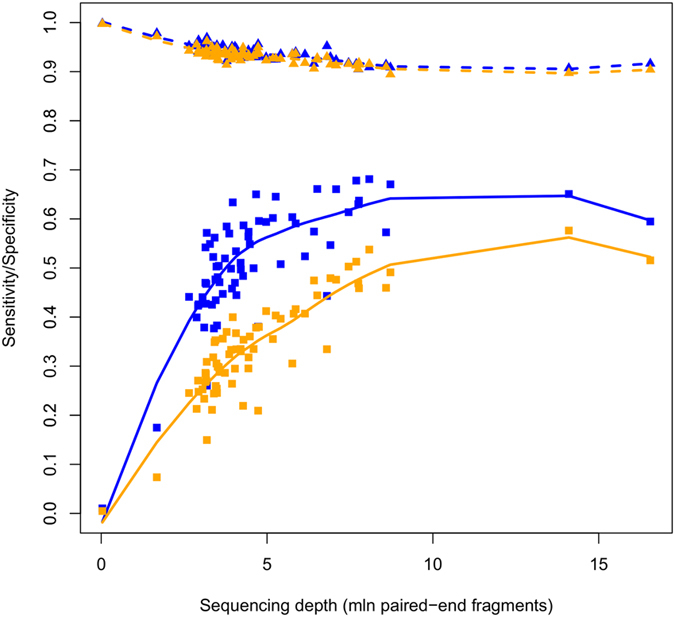
MethylCap-seq sensitivity reaches a plateau phase for increasing sequencing depths. Conditional sensitivity (squares) and specificity (triangles) are plotted as a function of sequencing depth, for both HM450 type 1 (blue) and type 2 (orange) assay types. Loess regression curves (using span smoothing parameter 0.95) have been added for both sensitivity (solid lines) and specificity (dashed lines) for both HM450 assay types.

Using a beta-value threshold of 0.3, Fig. 3 (includes 2 low coverage samples for graphical purposes) indicate that this is indeed the case. Of note, sensitivity reaches a plateau phase for sufficiently large sequencing depths. Indeed, a maximal sensitivity of about 65% (type I)/55% (type II assay loci) is obtained for sequencing depths larger than ~8 million fragments. As expected, specificity tended to decrease for higher sequencing depths, but stays clearly above 90%. For beta-value thresholds of 0.2 and 0.4 the same trends were observed, with a little lower (~5%) and marginally higher (~2%) maximal sensitivity values for thresholds 0.2 and 0.4, respectively, and specificities consistently above 90%.

To illustrate this methodological comparison in the context of a clinically highly relevant gene in glioblastoma, the binary MethylCap-seq and HM450 data were compared with calls obtained for the O6-methylguanine methyltransferase (*MGMT*) gene using methylation specific PCR (MSP)^30^. MSP data were available for 66 matching samples. The type I probe cg12981137 in the CpG island of *MGMT* interrogates a CpG comprised in the reverse primer of the MSP assay^31^, while encompassing an additional 5 CpGs, of which 2 are overlapping with the forward MSP primer. The kappa values were 0.76, 0.69 and 0.66 at cut–offs of 0.2, 0.3 and 0.4, respectively, for the HM450 probe and 0.48 for the matched MethylCap-seq site. In accordance with our previous results, a higher conditional sensitivity was observed for HM450, and a slightly higher conditional specificity for MethylCap-seq (no false positive vs 2, 1, and 0 for resp. HM450 cut-offs 0.2, 0.3 and 0.4) (see Supplementary Table S2).

### Quantitative and functional genome-wide features

Whereas MethylCap-seq exhibited a markedly lower sensitivity than HM450 for the loci assessed by the latter, this methodology is not limited by probes and can therefore interrogate more comprehensively “genome-wide” features. Therefore, it was evaluated to what extent methylated regions detected by MethylCap-seq were also assessed by HM450 probes. For each sample, MethylCap-seq methylated regions were identified as stretches of the genome featured by a positive number of mapped fragments separated by at least one nucleotide without mapped fragments.

The ratio of the number of probe loci with non-zero coverage and the total number of MethylCap-seq methylated regions for each sample thus provides an indication of the genome-wide characterization by HM450 vs. MethylCap-seq. This comparison revealed that only about 7.5% (median, IQR = [6.3%–9.8%]) of methylated regions identified by MethylCap-seq were also assessed by HM450. Of note, the use of ratios implicitly results in an adjustment for MethylCap-seq sequencing depth differences. Indeed, the ratios were not correlated with the sequencing depth (Spearman correlation, P = 0.42). Despite the detection of more methylated regions by MethylCap-seq, this does not automatically imply that also more relevant information will be retrieved.

Therefore, it was also evaluated to what extent the loci assessed by both methodologies differed with respect to CpG-island context and genomic functional location. For MethylCap-seq, the annotation of the center of the methylated regions was considered. Although no specific locations are targeted by MethylCap-seq, it should be noted that this approach is not completely unbiased due to the GC- and CpG-content dependent affinity of this method. Both for CpG-island context and genomic functional location, proportions of the features differed significantly between HM450, MethylCap-seq and their overall genomic distribution (Chi-square test, all P < 2.2E-16).

**Figure 4:**
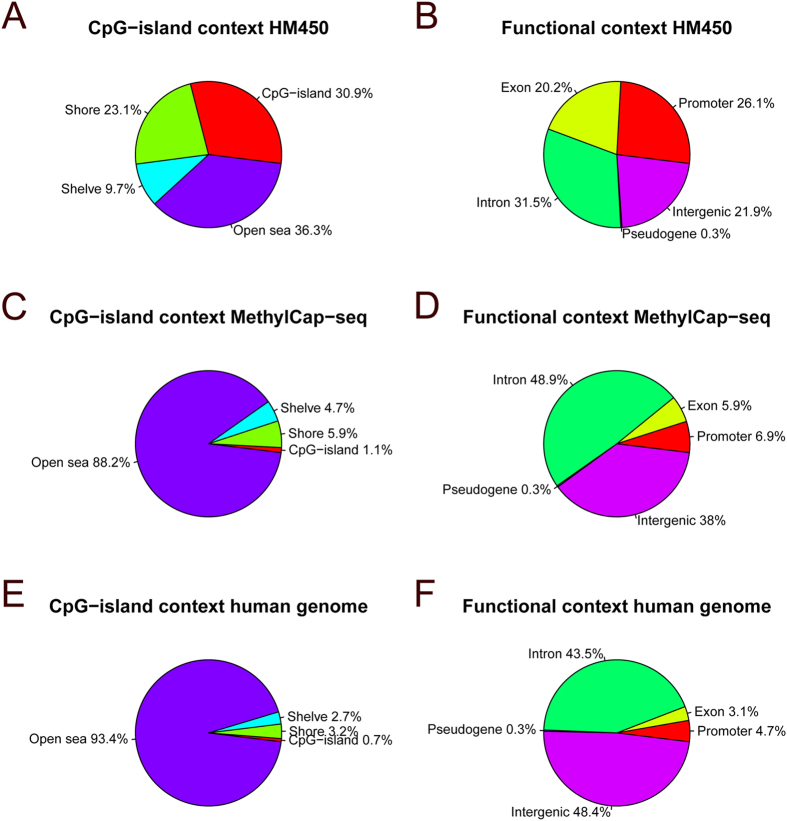
Fractions of CpG-island and functional location context assessed by both methodologies. Fractions of loci corresponding to CpG-islands, shores, shelves and open sea and fractions of promoter, exon, intron, pseudogene and intergenic loci assessed by HM450 (**A,B**) and detected by MethylCap-seq (**C,D**) and their genome-wide distribution (**E,F**).

This confirmed that the HM450 probe locations have specifically been selected towards CpG-islands and associated shores and shelves (Fig. 4A), as described elsewhere^14^. Also for MethylCap-based results (Fig. 4C), in comparison with the genome-wide occurrence of these features (Fig. 4E), there is apparent enrichment for CpG-islands, shores and shelves. For MethylCap-seq, variation in function of sequencing depth were very limited, with a slight decrease in CpG-island, shore and shelve fractions and an increase for the open sea fraction for higher sequencing depths (Supplementary Fig. S2, panel A), in line with the slightly decreasing specificity for increasing sequencing depths. When evaluating absolute numbers of detected methylated loci, using a beta-value threshold of 0.3 for HM450, the latter method detects more methylated loci in CpG-islands, but clearly less methylated loci in CpG-island shore and shelve regions (Table 2). Only considering MethylCap-seq samples with high sequencing depths did not alter these conclusions, nor did the application of other beta-value thresholds for presence of methylation (data not shown).

When compared with the distribution of genome-wide functional annotation (Fig. 4F), the HM450 predominantly targets promoters and exons (Fig. 4B), whereas MethylCap-seq data are enriched for the full length of the gene, including introns (Fig. 4D). Similar to the CpG-island context results, the proportion of intergenic regions picked up by MethylCap-seq increased with higher sequencing depths, whereas the different gene-associated functional genome annotations slightly decreased (except for the intron fraction, Supplementary Fig. S4, panel B). In absolute numbers, more methylated loci were detected for each functional annotation class by the MethylCap-seq methodology (Table 2), including promoter regions.

### Detection of differentially methylated loci

A comparison of methodologies may also include an assessment of the number of differentially methylated regions (DMRs). First, we assessed the intrinsic capacity of each methodology to detect potentially relevant loci, i.e. loci that were independently determined to be likely differentially methylated. Next, it was assessed whether this was also reflected in more significant differences between control and tumour samples in the dataset at hand.

As a proxy for “potentially relevant loci”, we evaluated to what extent HM450 and MethylCap-seq assess the set of genomic regions featured by dynamic DNA methylation, i.e. candidate DMRs (cDMRs) that were identified using 42 whole-genome bisulfite sequencing data sets by Ziller *et al.*^7^ Of the 485,512 cytosines targeted by HM450, no less than 160,481 (33.1%) can be found in such a cDMR. However, in total only 63,590 (8.9%) of the 716,087 cDMRs were covered by a HM450 assay (for at least one CpG). As not all cDMRs can be expected to be methylated in the sample set under consideration, only a lower limit estimation is possible for MethylCap-seq. On the other hand, considering all covered cDMRs would yield an overestimation due to the presence of non-specific signal. Taking into account the (minimal) specificity of 90% for MethylCap-seq in this study (see above), a CpG can be considered to be significantly methylated if fragments were picked up for this CpG in at least 12 out of 70 samples (significantly higher detection rate than 10% at α = 0.05, binomial distribution). Using this definition, significant methylation was picked up by MethylCap-seq in 353,873 (49.4%) of the cDMRs (for at least one CpG). Using a more stringent criterion, i.e. significant methylation in at least half of the CpGs in the cDMR, 170,384 (23.8%) of all cDMRs were identified as methylated, which is still clearly more than the potential of HM450.

Subsequently it was assessed whether this additional potential for MethylCap-seq also resulted in more significant differences between the 5 control and 63 glioblastoma samples. As in the previous section, two MethylCap-seq samples with lowest sequencing depths were not considered. For MethylCap-seq, the cDMRs identified by Ziller *et al.*^7^ were considered as variables. The same methodology was used for both datasets to enable an objective comparison. As outlined in the Methods section, dichotomized data (using 0.3 as cut-off for HM450) were compared using the Fisher exact test, and a permutation approach was used to obtain an estimate of the number of significant loci at a false discovery rate of 0.05.

This supervised analysis indicated that far more significant loci were found for HM450 (14,434) than for MethylCap-seq (233). Of the latter 233 regions, only 5 were targeted by one or more Infinium probes (9 probes in total), of which only one, cg21691166 (an intergenic probe targeting a locus near SLC14A1), was also significant by Fisher exact test analysis in the HM450 dataset (P = 0.009; Supplementary Tables S3 and S4). However, when performing a quantitative comparison for these 9 probes by the Welch t-test on the HM450 M-values, 7 out of 9 yielded a significant outcome (Supplementary Table S3), indicating that the selected data-analytical methodology (dichotomization procedure) rather than exact probe location or presence of noise and biases is the most important underlying cause of this apparent discrepancy. A comparison of the annotation for the significant loci indicated major differences in CpG and functional location context between HM450 and MethylCap-seq results (Supplementary Table S5), which however predominantly reflect the overall differences between both platforms as discussed above (Fig. 4). In line with the limited overlap of significant results between both platforms, numbers and fractions of significant results stratified per chromosome exhibited low similarity as well (Supplementary Table S6).

Additionally, it was assessed whether there was also a global difference between tumours and controls: upon principal coordinate analysis (PCO) of both binary datasets, permutations were used to infer the significance of this overall difference between both sample groups. Also here, this difference was present for both methodologies (Supplementary Fig. S5), but was more significant for HM450 (P < 0.001) than for MethylCap-seq (P = 0.009).

It is clear that in this particular experimental setting, both supervised and unsupervised results suggest that HM450 is able to detect more putatively relevant variation, i.e. discriminating between tumours and controls, than MethylCap-seq.

## Discussion

In this manuscript, MethylCap-seq and Infinium HM450 BeadChip data were compared for a large set of 70 brain tissue samples, mostly glioblastoma. The DNA used was obtained from the same DNA isolate for both methodologies. Overall, results were concordant, although this was to a lesser extent the case for Infinium type 2 assay loci, due to MethylCap-seq dependent biases. This is most likely related to the CpG- and GC-content of the broader probe target region, which might respectively affect the capturing and the sequencing steps of the MethylCap-seq procedure^22^,^23^,^24^. It is of note that MethylCap-seq sequencing depths were limited, we therefore focused on the detection of methylated loci rather than proceeding to a quantitative analysis, which is certainly a limitation of this study.

To assess conditional sensitivity and specificity for both methods, a Bayesian modelling procedure was used that does not rely on the comparison to a gold standard methodology. The latter would have been optimally suited for such an analysis yet was practically infeasible taking into account the large number of samples. It was clear that the sensitivity of MethylCap-seq to detect methylated loci was lower than for Infinium HM450, i.e. MethylCap-seq fails to detect a substantial part of methylated CpGs, and this was more prominent for only partially methylated alleles. Specificity was adequate for both methodologies, and higher for MethylCap-seq, although this difference was attenuated by higher MethylCap-seq sequencing depths. Sequencing depth also affected MethylCap-seq sensitivity, and we demonstrated that with approximately 8 million of fragments, maximal sensitivity regarding the detection of methylated loci is obtained. Importantly, this indicates that some methylated fragments are virtually impossible to capture or sequence by MethylCap-seq, even when one would use sequencing depths substantially higher than obtained in the here reported experiment. The previous comparison by Bock *et al.* already demonstrated (slightly) lower accuracy for MethylCap-seq than for HM27, which only contains type 1 assay loci^22^. Our results are in line with this observation for type 1 loci, but also show that sensitivity, and therefore overall accuracy, is even far lower for type 2 assay loci, most likely due to the specific sequence characteristics. As the sensitivity of MeDIP-seq also depends on CpG density, with inconclusive results whether this dependency is higher than for MethylCap-seq^32^,^33^, it is unlikely that this alternative enrichment based procedure might provide a far better solution.

We applied a modelling approach to estimate (conditional) sensitivity and specificity, which is a well substantiated strategy when an appropriate gold standard or reference is lacking^26^,^27^,^28^,^29^. In the large majority of the parameter estimation models, convergence was obtained, and results were overall very consistent. Moreover, in the population under study, overall conclusions were in line with MSP results for *MGMT* methylation, a highly relevant biomarker in glioblastoma^30^. This indicates that the Bayesian estimation approach provided indeed an appropriate yet affordable alternative for expensive, putatively “gold standard” methods such as RRBS. However, even with this alternative solution at hand, more extensive validation using an independent methodology remains to be the preferred strategy. One additional disadvantage is the need for considering methylation as a qualitative feature (absent/present).

Alternatively, quantitative assessment is possible for HM450 but also for MethylCap-seq. However, the sequencing depth was too limited in our study to fully exploit its potential, which is an important limitation of this study. On the other hand, from a practical point of view, in most studies DNA-methylation is considered as a qualitative phenomenon, imposing e.g. a minimal beta-value threshold for methylation, and using methylation specific PCR for validation purposes. Of note, for a quantitative approach to MethylCap-seq (or MeDIP-seq) based results, adjustment for locus-dependent biases (such as GC% and CpG-content) is absolutely necessary using algorithms such as BATMAN^34^.

Since shifts in parameter estimates (Table 1) were clearly larger between beta-value thresholds 0.2 and 0.3 than between 0.3 and 0.4, a threshold of 0.3 appears to be more suitable and this threshold was selected for the subsequent analyses. Although different thresholds might be appropriate for the two assay types due to the distinct beta-value distributions (cf. Fig. 1), a threshold of 0.3 appears to be a good consensus. Alternatively, data transformations can be used that increase overall correspondence between both HM450 assay type beta-value distributions or decrease overall technical artefacts^12^,^13^,^35^, but as raw data are already highly reproducible^16^ we elected to avoid data transformation and perform the analyses for both assay types separately.

Similarly, also other data preprocessing steps (e.g. probe filtering^15^, advanced background correction^36^, methylation status determination by mixture modeling^17^) and transformations (e.g. use of M-values^37^) and their combinations might improve results for HM450. Also here, we elected to avoid these often useful data modifications as these advanced methods either i) try to remediate inherent drawbacks of the methodology under evaluation, e.g. probe filtering^15^, ii) are not generally implemented (and often not independently validated), e.g. methylation state modeling, or iii) are not readily relevant for our goals, e.g. despite their less intuitive character, M-values are better for differential analysis of methylation levels^37^, but as these values are obtained by a monotonous transformation of β-values, β-value and M-value cut-offs yield equivalent results. Moreover, in all cases, results are clear to an extent that further data-analytical improvements will only have low impact. There are also experimental factors that could potentially have an impact on our conclusions regarding sensitivity and specificity. Indeed other potential sources of variation for the MethylCap-seq methodology are the capturing step of methylated fragments, i.e. the specific kit or methyl-binding domain used, as well as the salt concentration in the elution step. An initial evaluation suggested that the used MethylCap-seq kit yielded by the highest sensitivity and specificity, as independently evaluated by RRBS and HM27^38^. Also, as a single, high-salt elution was used for the MethylCap step, those fragments that are rather difficult to capture (e.g. featured by a lower CpG methylation degree) should have already been included. This implies that the used protocol already aimed for a maximal sensitivity. In addition, the difference in sensitivity between HM450 and MethylCap-seq regarding the detection of methylated loci was clear to an extent that it is very unlikely that different kits and validation methods would have a profound impact on this conclusion. Also, this is the first study performing a large-scale evaluation (70 samples) of both methodologies, providing major support to the reliability of our results, even when considering the limitations associated with the lack of a gold standard and the use of dichotomized data.

Sensitivity can also be considered from a “genome-wide” perspective, i.e. the number of loci identified to be methylated by a certain methodology. It is clear that MethylCap-seq has the inherent potential to detect far more methylated loci than the Infinium HumanMethylation BeadChip methodology, also in genomic regions that can be assumed to be biologically relevant (e.g. differentially methylated between case and control). However, in the current study, MethylCap-seq did not yield a better discrimination between both sample groups than HM450. In part, this can be attributed to the lower sensitivity of MethylCap-seq, implying that higher sequencing depths and a more balanced study design are required to obtain more significant results. The very large numbers of DMRs in combination with the clear PCO based separation of both sample groups for HM450 may also indicate that a clinically relevant fraction of glioblastoma samples are featured by a CpG island methylation phenotype (“G-CIMP”), leading to major global differences in methylation^39^. As MethylCap-seq is an enrichment based methodology, it is far less capable of detecting these global differences. Additionally, it should be noted that this would also imply violation of the independence assumption for false discovery rate estimation (see Methods), leading to an anti-conservative estimate of the number of significant genes, particularly for HM450. As a final remark in this comparison, although not directly related to here described results, it should be noted that a major advantage of MethylCap-seq over BeadChips is that the latter is human specific, whereas MethylCap-seq is more broadly applicable.

In conclusion, a comparative analysis revealed that HM450 and MethylCap-seq are rather complementary methods for the genome-scale detection of DNA-methylation. At least for this collection of brain samples (mostly glioblastoma), both exhibit high specificity, and while MethylCap-seq suffers from a lower sensitivity, it displays a far larger genome-wide potential for discovery. However, MethylCap-seq did not yield more DMRs in the study at hand, suggesting that more power and higher sequencing depths are required for MethylCap-seq to exploit this potential. Also, our results indicate that even with very high sequencing depths it is virtually impossible to profile all methylated loci in the human genome using MethylCap-seq. Additional validation, preferably featured by higher MethylCap-seq coverages and an independent gold standard methodology, is warranted to fully substantiate this claim.

## Methods

### Samples and DNA-isolation

DNA-methylation profiles were established for 65 glioblastoma tissues and 5 non-tumoral brain tissues (epilepsy surgery). All glioblastoma patients were treated within clinical trials (TMZ/RT pilot study^40^; EORTC Study 26981, www.clinicaltrials.gov, NCT#00006353^41^) and all subjects provided informed consent for molecular studies of their tissues, the trials conducted accruing to the approved good clinical practice guidelines and the Declaration of Helsinki; the clinical protocols were approved by the ethics committees at each participating center and the respective competent authorities. The use of biological material was approved by the Ethics committee of Canton of Vaud, Lausanne, Switzerland (approval F25/99/ Commission cantonale d'étique de la recherche sur l'être humain, canton de Vaud, Lausanne Switzerland).

Description of the samples has been published previously^40^,^41^,^42^. Methods have been carried out in accordance with approved guidelines. DNA was isolated from snap frozen samples using a genomic DNA isolation kit including an RNase treatment step (Qiagen kit 19060). DNA was obtained from the same isolate for the HM450 and MethylCap-seq assays, implying no differences due to tumour heterogeneity.

### HM450 assay

For each sample, 1.0 μg of DNA was bisulfite converted using the EZ DNA Methylation Kit (Zymo Research) and analyzed on HM450 BeadChips at the University of Gevena’s Genomics platform, both according to the manufacturers’ instructions. The Illumina GenomeStudio program was used for normalization and extraction of the methylated and unmethylated signal intensities. The probes’ characteristics were derived from the *IlluminaHumanMethylation450k.db* Bioconductor package. Probes without annotation (chromosome/locus) were removed, yielding a final dataset consisting of 485,512 assessed loci. For all samples, detection P-values were lower than 0.05 for over 99.9% of all loci; non-significant loci (P > 0.05) were not considered in the subsequent analysis. There is no consensus regarding beta-value cut-off values for presence of methylation, although typically thresholds of 0.2 and 0.3 are used^37^,^43^. To take into account variation of this arbitrary threshold, a range of 4 cut-offs (0.1 to 0.4) was used. However, as the 0.1 threshold was clearly too low to obtain reliable results, data for this threshold have been omitted from this manuscript.

### MethylCap-seq

A full description of the MethylCap-seq procedure can be found in De Meyer *et al.*^38^, with minor modifications as mentioned *infra*. In summary, 500 ng of DNA per sample was fragmented by ultrasound mediated DNA shearing (Covaris S2, Covaris, Woburn, Massachusetts, USA) set to obtain fragments with an average length of 200 bp. We employed the MethylCap kit (Diagenode AF-100-0048, Liège, Belgium) to obtain methylated DNA fragments using the highest salt concentration single elution step option. This was followed by the library preparation for the enriched DNA, which was performed with a modified ‘multiplexed paired-end ChIP protocol’ (Illumina, San Diego, California, USA). Therefore, the NEBNext® DNA Sample Prep Master Mix Set 1 (New England BioLabs (NEB) E6040, Ipswich, Massachusetts, USA) was combined with the Multiplexing Sample Preparation Oligo Kit (Illumina PE-400–1001). Per sample, 22 μl of the prepared sample was subjected to PCR (21 cycles) following the Illumina Library Amplification Index Protocol. After purification (Qiaquick PCR Purification columns, Qiagen), elution and concentration (evaporation), the libraries were pooled and denaturated (NaOH), followed by Illumina GAIIx sequencing (‘multiplexed paired-end run, 2 times 45 cycles) of 10pM/pool.

Sequence data was processed with an in house developed pipeline using Perl 5.12.2. Paired-end reads were mapped using BOWTIE^44^, and only fragments for which reads mapped uniquely within a 400bp of each other in the human reference genome (NCBI build 37) were retained. Fragments for which paired-end reads mapped on exactly the same locations were considered as PCR duplicates and further treated as a single fragment. Loci were considered to be methylated if at least one fragment could be mapped on that position.

### ENCODE data

For independent validation of observed phenomena, RRBS and HM450 data generated by the ENCODE consortium^25^ were downloaded for cell lines HCT-116, Caco-2 and MCF-7, originating from Stanford University, University of Washington and Duke University (http://encodeproject.org/ENCODE/downloads.html, replicate 1 for each cell line), respectively. Following the ENCODE recommendations, only CpGs with a minimal RRBS coverage of 10 were included in the analysis. For HCT-116, Caco-2 and MCF-7, respectively 18,168, 40,782 and 31,183 loci were identified that overlapped between both methodologies.

### Bayesian modeling to estimate conditional sensitivity and specificity

Uncertainty related to the choice of the threshold for dichotomization of the HM450 data and the presence of other biases also implies that neither platform can be considered as gold standard for the definition of the methylation status of a given CpG. Consequently, a Bayesian modeling procedure was used to estimate the median of the sensitivity, specificity and the true prevalence for each sample and each beta threshold. We used the alternate parameterization proposed by Dendukuri and Joseph^27^, and used by de Clare Bronsvoort and coworkers^28^, for the two dependent tests, one population scenario. The model, based on a multinomial likelihood function, involves the estimation of seven parameters: sensitivity and specificity for each method, methylation prevalence and covariances between the two methods among the methylated and unmethylated CpG. The implementation and properties of this model have been described in detail by Dendukuri and Joseph^27^ and Branscum *et al.*^26^ Because the model was non-identifiable, the initial conditions were redefined with different prior values for each Markov Chain. Besides visual inspection of the MCMC chain, we computed the Gelman and Rubin 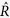 statistic as convergence measure^29^. 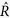 provides an easy–to–interpret measure of whether independent chains have converged to sample from the same distribution. After convergence, 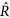 should be very close to 1 (as a rule of thumb, smaller than 1.1) for all parameters indicating good mixing of the chains and thus approximate convergence. The Bayesian model was performed in the freeware program WinBUGS^45^ (version 1.4.3) and R package R2WinBUGS^46^. The estimations were obtained after 200,000 iterations for five Markov chains with different starting positions, of which only the second half of the iterations was considered to be representative of the stationary distribution of the chains, in order to obtain accurate inference from the Gibbs sampler.

### Loess regression and functional annotation information

Additional data-analysis was performed in the R statistical environment, version 2.15.2. For loess regression, the R “stats” package function “loess” was used. CpG-island annotation was downloaded from UCSC (http://hgdownload.cse.ucsc.edu/, latest version - April 13, 2006). Using common definitions, e.g. by Pan and colleagues^11^, CpG-island shores were defined as those regions up to 2 kb up- or downstream from the CpG-island start and end, respectively. The regions another 2 kb up- or downstream from the shore boundaries were defined as CpG-island shelves. The remaining fraction of the genome was annotated as “open sea”.

Functional genomic information was downloaded from Ensembl’s Biomart database (accessed on January 9, 2013). As we aimed at a comparison between methodologies, relatively arbitrary definitions could be used. Promoter regions were defined as those regions 1.5 kb upstream to 0.5 kb downstream of the transcription start site (taking into account direction of the transcript) of all transcripts in the database. All regions annotated as such were considered as exon, except for the parts overlapping with promoter regions. Other regions within the gene boundaries can generally be considered as intronic, and were annotated as such. The pseudogene annotation by Ensembl was followed, but regions overlapping with non-pseudogenes were truncated.

### Detection of differentially methylated regions and global methylation differences

Differentially methylated regions were assessed by the Fisher exact test on the dichotomized data sets (beta-value cut-off of 0.3 for HM450), considering solely those loci that were not consistently methylated or unmethylated in all samples under study (162,872 loci for HM450, 660,983 for MethylCap-seq; two samples with lowest MethylCap-seq sequencing depths were not included in these analyses). As most false discovery rate estimation strategies use assumptions that are not valid for these non-continuous data, a permutation based approach was used to obtain a P-value distribution under the null hypothesis.

For each locus in the data set under study individually, class labels were permuted, leading to a randomized dataset that should contain virtually no association with the phenotype, but still has the same average methylation frequency per locus. Subsequently, upon ranking of the P-values for the actual dataset, for each P-value, P_i_ – with i ranging from 1 to the number of loci considered, a corresponding false discovery rate can be calculated as the number of null P-values smaller than or equal to P_i_ divided by i. Note that this approach is based on the conservative assumption that the fraction of non-null loci equals 0 and that it ignores dependency between variables.

An alternative approach is to evaluate to which extent the major sources of variation in each dataset are associated with the differences between case and control samples. Therefore, for each dataset, principal coordinate analysis (PCO)^47^ was performed on the Jaccard distances between samples^48^. A Monte-Carlo test (1000 permutations) on between-group inertia (global test) was performed to test the overall difference between tumor and control samples^49^. PCO, between-group tests and graphical representations were performed using the “ade4” R package with standard options^50^.

## Additional Information

**Accession codes**: Infinium HumanMethylation450k BeadChip data has been deposited in GEO (NCBI) under accession number GSE60274 and is freely available. Raw MethylCap-seq data has been submitted to EGA (EBI, EGAS00001001191) and can be accessed upon approval by the Data Access Committee.

**How to cite this article**: De Meyer, T. *et al.* Genome-wide DNA-methylation detection by MethylCap-seq and Infinium HumanMethylation450 BeadChips: an independent large-scale comparison. *Sci. Rep.* **5**, 15375; doi: 10.1038/srep15375 (2015).

## Supplementary Material

Supplementary Information

## Figures and Tables

**Table 1:** Gold standard independent DNA-methylation prevalence estimation, and sensitivity and specificity estimation for HM450 and MethylCap-seq.

Beta	Stat.	Method	Type 1 chemistry loci			Type 2 chemistry loci		
			*Mean (SD)*	*Min.*	*Max.*	*Mean (SD)*	*Min.*	*Max.*
0.2	Prev.	–	54.0 (3.3)	48	60.9	79.5 (1.5)	75.6	83.0
	Sens.	HM450	76.8 (2.1)	71.6	80.9	91.3 (0.9)	89.3	93.6
	Spec.	HM450	85.7 (1.0)	83	87.6	80.3 (0.8)	78.3	82.1
	Sens.	MCap	50.1 (9.2)	24.7	67.1	32.8 (8.7)	14.1	52.0
	Spec.	MCap	94.1 (1.3)	90.6	97.1	93.4 (1.1)	90.9	95.3
0.3	Prev.	–	51.5 (3.5)	44.5	60.0	75.3 (2.1)	69.6	78.5
	Sens.	HM450	74.3 (2.5)	66.5	79.0	89.5 (1.2)	87.2	92.1
	Spec.	HM450	86.8 (1.1)	84.5	89.5	81.0 (0.9)	79.2	83.7
	Sens.	MCap	52.5 (9.0)	26.0	68.1	34.5 (9.3)	15.0	57.6
	Spec.	MCap	93.8 (1.4)	90.5	96.9	93.1 (1.4)	89.5	96.2
0.4	Prev.	–	49.4 (3.9)	41.1	59.1	71.2 (2.9)	63.0	77.5
	Sens.	HM450	71.8 (2.6)	65.2	77.5	87.8 (1.4)	84.2	90.9
	Spec.	HM450	87.7 (1.2)	85.4	90.4	81.6 (0.9)	79.7	83.8
	Sens.	MCap	53.9 (8.5)	27.1	68.5	35.8 (9.4)	15.6	59.8
	Spec.	MCap	93.1 (1.7)	88.8	96.7	92.8 (1.5)	89.3	95.7

Data indicate mean (standard deviation, SD) and minimum (min.) and maximum (max.), over the different samples, for different statistics (stat.) for both methods (MCap indicates MethylCap-seq): methylation prevalence (prev.), sensitivity (sens.) and specificity (spec.) for both chemistries and different HM450 beta-value thresholds (Beta) for presence of methylation. Models presenting strong convergence problem and two samples with very low coverage (samples 38 and 43, Supplementary Table 1) were not considered in the estimation of the statistics. Nevertheless, for each statistic at least 90% of the samples were used.

**Table 2:** Comparison of the numbers of methylated loci, stratified by functional annotation, between HM450 and MethylCap-seq.

Feature	HM450	MethylCap-seq	Sign. diff.
CpG-island context			
CpG-islands	44,480 [41,357–47,161]	17,679 [15,310–21,139]	3.6E-13
Shores	71,157 [69,366–72,560]	95,552 [85,317–111,682]	2.5E-10
Shelves	44,262 [43,576–44,666]	78,239 [68,305–92,023]	8.9E-12
Open sea	155,028 [150,657–157,864]	1,495,205 [1,140,952–1,885,495]	4.0E-13
Functional genomic location context			
Promoters	64,759 [63,112–66,207]	113,817 [96,797–137,230]	9.2E-12
Exons	62,123 [61,246–63,027]	97,737 [85,470–115,403]	2.4E-11
Introns	115,779 [113,247–117,296]	817,975 [649,211–1,026,661]	4.0E-13
Pseudogenes	1,058 [1,048–1,070]	4,512 [3,637–5,384]	4.3E-13
Intergenic	71,120 [69,181–72,292]	643,160 [479,131–816,080]	4.0E-13

Data indicate median [IQR (interquartile range)] numbers of methylated loci detected for the 70 samples, significance of the differences (sign. diff.) between absolute numbers for both methodologies was evaluated with the Kruskal-Wallis signed rank test. A beta-value threshold of 0.3 was used for presence of methylation for HM450.
