# Supplementary material for: Genome-wide DNA methylation detection by MethylCap-seq and Infinium HumanMethylation450 BeadChips: an independent large-scale comparison
Source: Sci Rep. 2015 Oct 20;5:15375. doi: 10.1038/srep15375 (PMC4612737; doi:10.1038/srep15375)
Supplement: Supplementary Information [file srep15375-s1.pdf]

Supplementary Information

Genome-wide DNA methylation detection by MethylCap-seq and Infinium HumanMethylation450 BeadChips: an independent large-scale comparison

[1]Tim De Meyer, [2,3,4]Pierre Bady, [1]Geert Trooskens, [2,3,5]Sebastian Kurscheid[$], [5]Jocelyne Bloch, [6]Johan M. Kros, [7]Johannes A. Hainfellner, [5,8]Roger Stupp, [3,4]Mauro Delorenzi, [2,5]Monika E. Hegi, [1]Wim Van Criekinge.

[1]Dept. of Mathematical Modelling, Statistics and Bioinformatics, Faculty of Bioscience Engineering, Ghent University, Ghent, Belgium

[2]Lab. of Brain Tumor Biology and Genetics, Department of Clinical Neurosciences, University Hospital Lausanne, Lausanne, Switzerland

[3]Swiss Institute of Bioinformatics, Lausanne, Switzerland

[4]Département de Formation et Recherche, University Hospital / University of Lausanne, Lausanne, Switzerland

[5]Neurosurgery, Department of Clinical Neurosciences, University Hospital Lausanne, Lausanne, Switzerland

[6]Division of Neuropathology, University Hospital Rotterdam, Rotterdam, The Netherlands

[7]Institute of Neurology, Medical University of Vienna, Vienna, Austria

[8]Department of Oncology, University Hospital Zurich, Zurich, Switzerland

[$]Current affiliation: The John Curtin School for Medical Research, The Australian National University, Canberra, Australia

Supplementary Table S1 – HM450 BeadChip and MethylCap-seq data summary

Sample number	Sample ID	HM450 excluded loci	HM450 beta-value	HM450 intensity	MCap seq. depth	MCap coverage
1	1076	169 (0.0348%)	0.536 (0.349)	29,109 (12,366)	5,863,034	0.792 (4.906)
2	1297	66 (0.0135%)	0.483 (0.334)	28,100 (12,634)	3,444,194	0.453 (4.641)
3	1308	305 (0.0628%)	0.529 (0.341)	24,118 (10,253)	4,075,239	0.456 (3.062)
4	1710	104 (0.0214%)	0.565 (0.348)	24,784 (11,233)	3,780,961	0.588 (1.461)
5	1357	92 (0.0189%)	0.597 (0.367)	25,611 (10,352)	7,689,183	1.668 (4.499)
6	1360	120 (0.0247%)	0.519 (0.350)	26,751 (11,485)	2,934,773	0.320 (1.465)
7	1399	276 (0.0568%)	0.519 (0.338)	27,924 (13,044)	7,771,091	1.022 (3.339)
8	1714	101 (0.0208%)	0.554 (0.346)	28,204 (11,733)	3,730,539	0.451 (1.397)
9	1430	82 (0.0168%)	0.513 (0.342)	24,155 (11,180)	3,474,325	0.449 (1.519)
10	1437	105 (0.0216%)	0.535 (0.341)	23,545 (10,422)	3,860,504	0.555 (1.665)
11	1496	104 (0.0214%)	0.588 (0.362)	24,557 (10,310)	4,290,218	0.629 (1.616)
12	1497	81 (0.0166%)	0.499 (0.336)	26,305 (12,082)	4,438,217	0.649 (3.207)
13	1543	106 (0.0218%)	0.497 (0.338)	30,462 (13,738)	3,168,903	0.363 (1.234)
14	1545	84 (0.0173%)	0.523 (0.341)	26,240 (11,099)	3,665,563	0.474 (4.362)
15	1552	70 (0.0144%)	0.515 (0.342)	27,215 (10,936)	4,063,263	0.545 (2.689)
16	1562	351 (0.0722%)	0.516 (0.345)	24,737 (9,926)	6,513,537	1.247 (4.481)
17	1564	62 (0.0127%)	0.496 (0.338)	29,124 (11,914)	7,081,936	1.245 (3.565)
18	1566	391 (0.0805%)	0.511 (0.343)	27,031 (11,614)	3,490,278	0.309 (1.624)
19	1584	77 (0.0158%)	0.552 (0.361)	23,720 (10,677)	4,755,629	0.705 (1.977)
20	1587	360 (0.0741%)	0.532 (0.349)	26,902 (11,418)	3,279,593	0.567 (1.843)
21	1589	98 (0.0201%)	0.524 (0.338)	26,372 (11,421)	3,485,154	0.382 (1.360)
22	1591	100 (0.0205%)	0.539 (0.350)	24,612 (11,028)	4,443,768	0.639 (1.642)
23	1704	85 (0.0175%)	0.465 (0.325)	25,647 (11,265)	3,943,860	0.343 (1.045)
24	1603	69 (0.0142%)	0.543 (0.355)	25,693 (11,484)	7,458,910	0.990 (5.326)
25	1606	335 (0.0689%)	0.515 (0.344)	23,325 (10,365)	8,082,158	1.640 (5.462)
26	1612	180 (0.0370%)	0.515 (0.337)	23,449 (12,290)	3,185,882	0.169 (1.141)
27	1613	98 (0.0201%)	0.511 (0.341)	24,953 (11,807)	4,031,830	0.385 (2.576)
28	1730	84 (0.0173%)	0.499 (0.349)	26,412 (11,262)	3,181,935	0.579 (1.625)
29	1617	61 (0.0125%)	0.524 (0.343)	26,278 (10,700)	4,669,502	1.312 (4.084)
30	1622	47 (0.0096%)	0.555 (0.335)	28,211 (11,173)	3,418,966	0.623 (1.819)
31	1628	363 (0.0747%)	0.535 (0.349)	26,228 (10,483)	3,143,970	0.507 (1.185)
32	1639	164 (0.0337%)	0.527 (0.336)	28,752 (11,978)	4,593,961	0.452 (1.668)
33	1647	351 (0.0722%)	0.510 (0.331)	30,179 (12,628)	4,972,501	0.727 (1.955)
34	1703	89 (0.0183%)	0.554 (0.347)	27,307 (12,143)	3,966,636	0.848 (2.036)
35	1614	120 (0.0247%)	0.528 (0.355)	23,362 (10,955)	4,481,043	0.568 (1.629)
36	1705	116 (0.0238%)	0.549 (0.358)	30,546 (12,899)	5,261,747	0.812 (1.891)
37	1706	51 (0.0105%)	0.533 (0.336)	25,047 (10,191)	5,176,570	0.725 (1.606)
38	1419	380 (0.0782%)	0.518 (0.333)	22,566 (12,003)	1,676,347	0.084 (0.353)
39	1316	71 (0.0146%)	0.521 (0.348)	24,799 (10,431)	4,731,366	0.264 (1.073)
40	1601	336 (0.0692%)	0.505 (0.332)	24,623 (10,908)	5,761,703	0.610 (1.538)
41	1731	79 (0.0162%)	0.499 (0.346)	26,862 (11,498)	4,277,480	0.369 (1.131)
42	1759	83 (0.0170%)	0.522 (0.341)	25,880 (11,393)	3,141,112	0.384 (1.352)
43	1756	45 (0.0092%)	0.549 (0.357)	24,593 (10,389)	35,271	0.005 (0.072)

44	2901	65 (0.0133%)	0.514 (0.351)	26,777 (11,406)	3,397,695	0.289 (2.135)
45	1752	73 (0.0150%)	0.551 (0.346)	28,611 (11,533)	16,554,961	1.072 (4.523)
46	2683	113 (0.0232%)	0.510 (0.346)	24,593 (10,913)	3,340,906	0.294 (0.894)
47	2669	84 (0.0173%)	0.518 (0.342)	25,766 (10,715)	2,877,157	0.272 (0.777)
48	2540	93 (0.0191%)	0.524 (0.348)	25,921 (10,755)	3,056,287	0.376 (1.123)
49	2207	72 (0.0148%)	0.488 (0.334)	22,140 (10,368)	4,209,976	0.447 (1.435)
50	1780	51 (0.0105%)	0.518 (0.344)	26,973 (11,335)	6,805,473	0.454 (2.470)
51	1775	127 (0.0261%)	0.530 (0.351)	23,133 (9,882)	5,802,322	0.728 (2.881)
52	1774	126 (0.0259%)	0.558 (0.353)	27,078 (11,271)	6,143,336	0.631 (3.455)
53	1769	152 (0.0313%)	0.548 (0.343)	27,860 (11,383)	5,411,432	0.588 (3.668)
54	1768	352 (0.0725%)	0.501 (0.334)	25,938 (10,972)	8,588,190	0.798 (2.906)
55	1766	75 (0.0154%)	0.528 (0.338)	29,707 (12,919)	4,203,162	0.484 (2.505)
56	1764	60 (0.0123%)	0.543 (0.357)	24,138 (10,472)	8,719,737	1.005 (3.216)
57	1763	104 (0.0214%)	0.525 (0.352)	24,860 (11,838)	3,564,916	0.394 (1.461)
58	1761	75 (0.0154%)	0.544 (0.365)	23,415 (10,301)	3,377,179	0.472 (1.602)
59	1758	88 (0.0181%)	0.506 (0.345)	23,347 (10,437)	3,914,229	0.451 (1.716)
60	2903	378 (0.0778%)	0.546 (0.344)	26,988 (11,081)	3,453,319	0.358 (1.512)
61	1755	302 (0.0622%)	0.538 (0.348)	26,670 (11,324)	14,098,604	1.277 (10.652)
62	2900	357 (0.0735%)	0.535 (0.346)	27,754 (11,726)	3,105,763	0.293 (1.342)
63	1749	270 (0.0556%)	0.529 (0.339)	26,308 (10,598)	3,545,171	0.442 (1.220)
64	1748	58 (0.0119%)	0.548 (0.362)	23,940 (10,568)	3,511,806	0.425 (1.466)
65	1732	373 (0.0768%)	0.562 (0.349)	25,470 (10,693)	2,921,152	0.363 (1.500)
66	1729	106 (0.0218%)	0.494 (0.344)	30,402 (13,573)	7,751,982	0.891 (3.037)
67	1709	94 (0.0193%)	0.508 (0.353)	25,398 (11,359)	2,647,993	0.330 (1.274)
68	1707	318 (0.0654%)	0.510 (0.340)	26,640 (10,889)	3,156,515	0.375 (1.707)
69	1621	72 (0.0148%)	0.558 (0.353)	25,055 (9,965)	6,911,561	0.731 (5.002)
70	1317	62 (0.0127%)	0.504 (0.334)	24,536 (10,799)	6,414,966	0.801 (6.436)

Sample number is used throughout manuscript, sample ID refers to identifiers used in data repositories (EGA and GEO). HM450 Excluded loci indicates the number (and percentage) of loci featured by a detection P-value > 0.05, of the total of 485,512 loci considered. HM450 beta-value and intensity indicate the average (standard deviation) beta-values resp. intensities (A + B probes) over all assessed loci for that sample (Infinium HM450 BeadChips); MCap seq. depth indicates the number of uniquely mapped fragments generated by MethylCap sequencing for that sample (with 2 sequences per fragment, due to the paired-end sequencing); MCap coverage indicates the average (standard deviation) MethylCap-seq coverage values for those loci assessed by Infinium HM450 BeadChips.

Supplementary Table S2 – DNA methylation comparison between MSP, HM450 and MethylCap-seq for the locus interrogated by cg12981137 (MGMT)

Platform	Cut-off	Status	U (MSP)	M (MSP)	Kappa (MSP)	Kappa (MCap/HM450)
HM450	0.2	U	32	6	0.7565	0.5045
		M	2	26		
	0.3	U	33	9	0.6944	0.5372
		M	1	23		
	0.4	U	34	11	0.6630	0.5464
		M	0	21		
MCap		U	34	17	0.4762	
		M	0	15		

Count data reflect methylation status (U – unmethylated; M – methylated) as measured by Methylation Specific PCR (MSP) and compared with respectively Infinium HM450 BeadChips (HM450) and MethylCap-seq (MCap). For HM450, three different cut-offs (0.2, 0.3, 0.4) were used for beta-value dichotomization, as outlined in the main article. Concordance between MSP results and respectively HM450 and MCap is evaluated by the Kappa index, Kappa (MSP). Kappa (MCap/HM450) reflects the concordance between MCap and HM450 (for different HM450 cut-offs, independent of MSP results) for this locus.

Supplementary Table S3 – HM450 outcome for significant MethylCap results targeted by HM450 probes

Chrom.	Start	End	ProbeID	Nearest gene (Func. loc.)	Fisher P	t-test P
1	182,809,473	182,810,798	cg26546113	DHX9 (5'UTR)	0.578	0.197
5	95,453,420	95,454,541	cg14111437	MIR583 (Intergenic)	1	0.078
11	7,259,144	7,259,475	cg06712088	SYT9 (Intergenic)	1	8.6E-9
11	7,259,144	7,259,475	cg20653032	SYT9 (Intergenic)	1	2.1E-6
18	43,355,234	43,356,159	cg16245698	SLC14A1 (Intergenic)	1	9.8E-3
18	43,355,234	43,356,159	cg18088442	SLC14A1 (Intergenic)	0.055	4.9E-8
18	43,355,234	43,356,159	cg21691166	SLC14A1 (Intergenic)	0.009	3.6E-6
18	43,355,234	43,356,159	cg25416153	SLC14A1 (Intergenic)	0.058	2.4E-4
20	17,115,552	17,115,823	ch.20.356846R	PCSK2 (Intergenic)	1	1.5E-3

Data represent chromosome (chrom.), start and end position of Ziller regions that were significant upon Fisher exact test analysis of MethylCap-seq data (tumor samples vs. controls) and that were also targeted by Infinium HM450 probes. For the latter, probe identifier (ProbeID), symbol of nearest gene (according to UCSC Genome Browser on GRCh37/hg19 assembly) and functional location (Func. loc.) are presented as well as Fisher exact test (dichotomized data) and Welsh t-test (quantitative M-value data) P-values.

Supplementary Table S4 – Comparison of MethylCap-seq and HM450 (cg21691166) results for the locus detected as significant by both methodologies (*cf.* Table S3)

Controls (N = 5)		
	MethylCap-seq	
HM450	Methylated	Unmethylated
Methylated	0	0
Unmethylated	0	5
Tumours (N = 63)		
	MethylCap-seq	
HM450	Methylated	Unmethylated
Methylated	35	5
Unmethylated	20	3

Data represent contigency tables of methylation status as assessed by both methodologies separately for control and tumour samples

Supplementary Table S5 – Significant differences stratified by CpG and functional location context annotation for both methodologies

Annotation	MethylCap (N=233)	MethylCap (%)	HM450 (N=14434)	HM450 (%)
CpG context				
CpG-island	1	0.43	5,134	35.57
Shore	6	2.58	3,493	24.20
Shelve	6	2.58	748	5.18
Open sea	222	95.28	5,059	35.04
Functional location				
Promoter	12	5.15	3,389	23.48
Exon	13	5.58	2,511	17.40
Intron	86	36.91	4,347	30.12
Pseudogene	1	0.43	22	0.15
Intergenic	129	55.36	4,165	28.86

Data represent numbers and percentages of significantly differentially methylated loci stratified by CpG and functional location context. For MethylCap-seq, the used regions (Ziller cDMRs) may overlap with multiple elements, explaining why summed percentages exceed 100%.

Supplementary Table S6 – Number of significant differences stratified by chromosome for both methodologies

Chromosome	MethylCap	MethylCap (%)	HM450	HM450 (%)
1	12	5.15	1309	9.07
2	19	8.15	1248	8.65
3	22	9.44	677	4.69
4	17	7.30	627	4.34
5	23	9.87	873	6.05
6	13	5.58	1193	8.27
7	6	2.58	1092	7.57
8	14	6.01	661	4.58
9	15	6.44	283	1.96
10	17	7.30	595	4.12
11	15	6.44	751	5.20
12	10	4.29	770	5.33
13	15	6.44	344	2.38
14	7	3.00	453	3.14
15	8	3.43	445	3.08
16	7	3.00	542	3.76
17	1	0.43	739	5.12
18	7	3.00	218	1.51
19	1	0.43	604	4.18
20	3	1.29	328	2.27
21	1	0.43	142	0.98
22	0	0.00	209	1.45
X	0	0.00	319	2.21
Y	0	0.00	12	0.08

Supplementary Figures

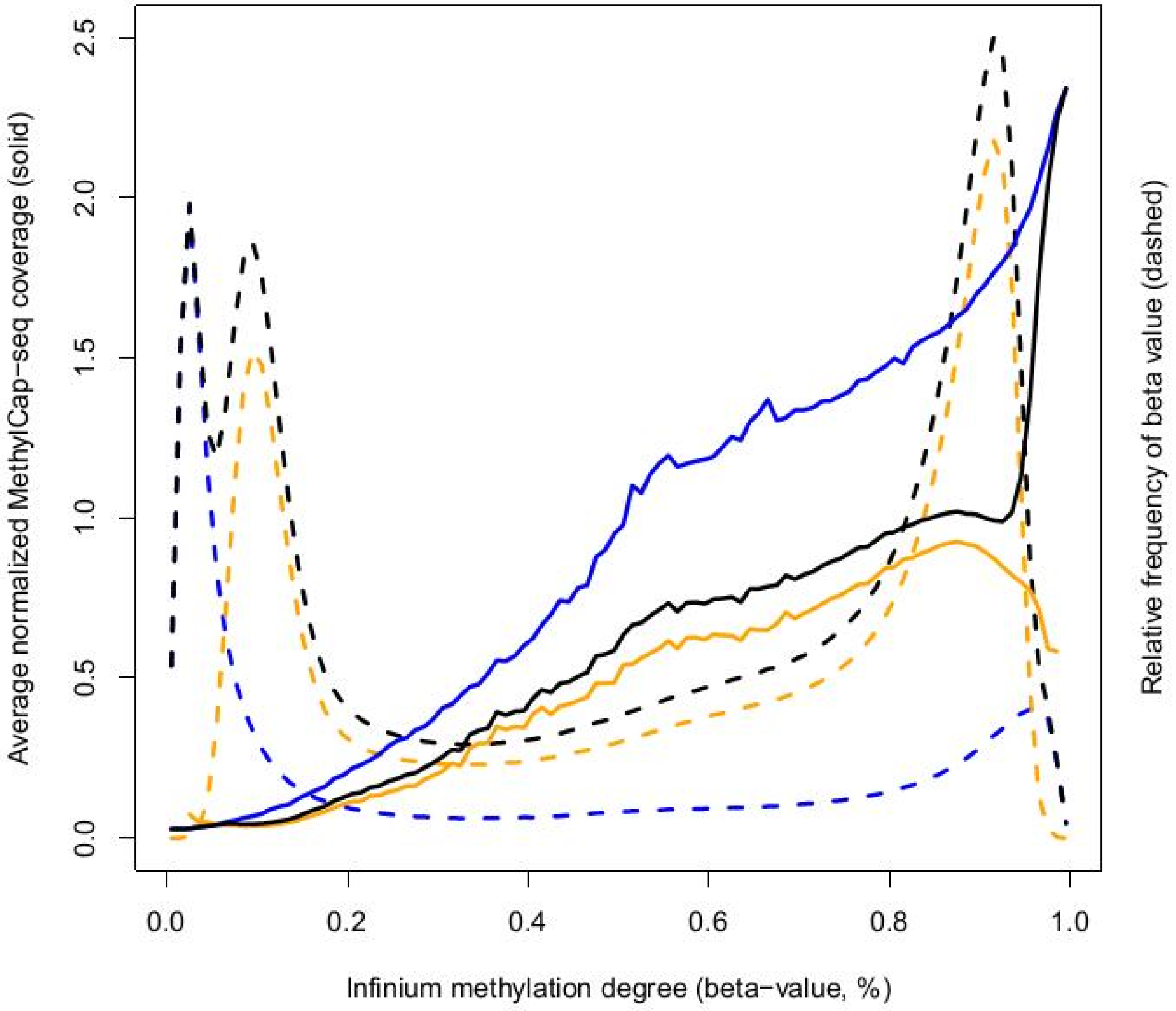

Figure S1 - Concordance between MethylCap-seq (continuous) and Infinium HM450 BeadChip results. The average MethylCap-seq coverage values (over all samples, solid lines) are plotted as a function HM450 methylation degrees (beta-values, binned per 1%), for both types of HM450 assays: type 1 (blue), type 2 (orange), and combined (black). The beta-value distributions are depicted as dashed lines for both types individually (type 1, blue; type 2, orange) and combined (black). MethylCap-seq values were normalized using library size normalization (= to the average library size).

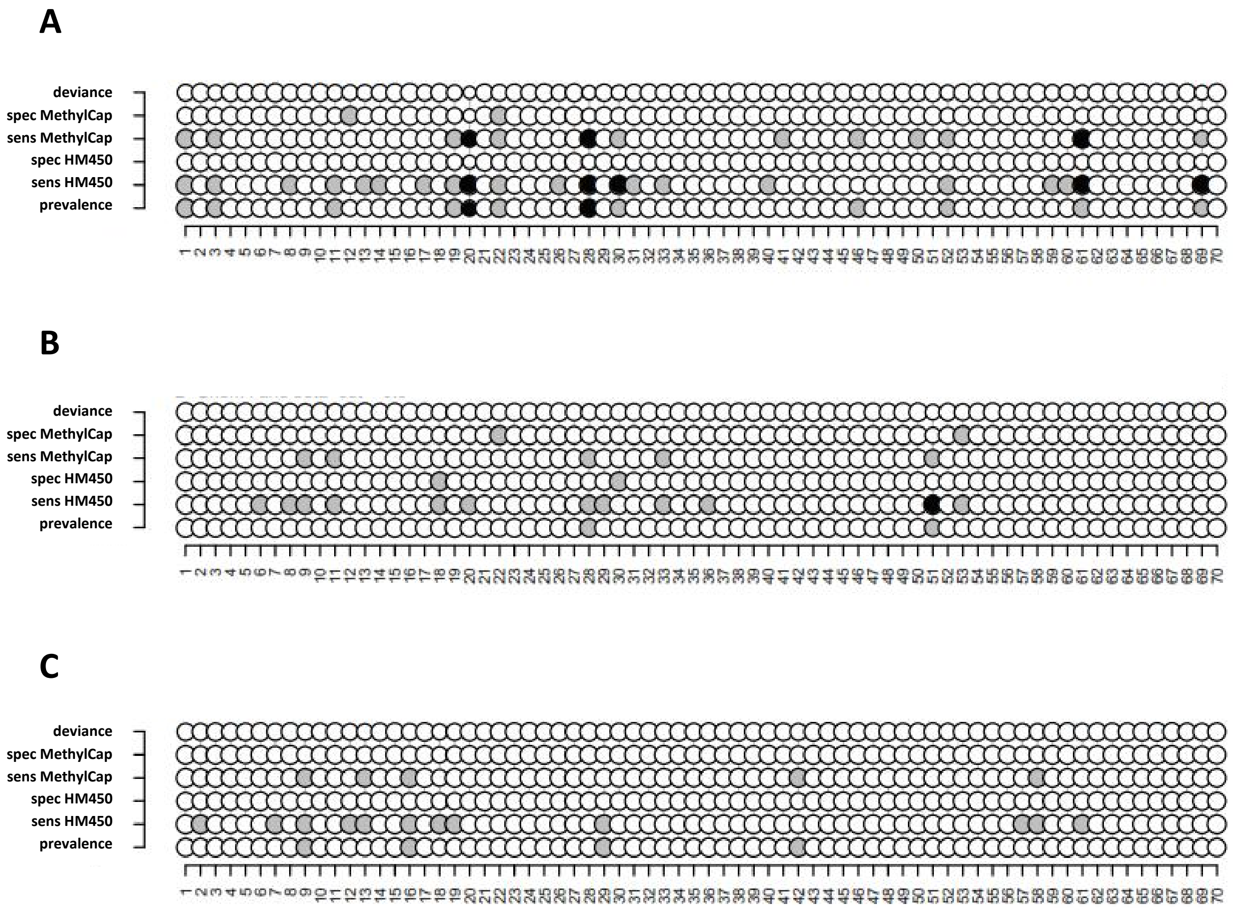

Figure S2 – Model convergence regarding deviance, prevalence, sensitivity and specificity estimation for assay type 1 loci. For each sample (X-axis), convergence results regarding deviance, specificity (spec), sensitivity (sens) and prevalence for both MethylCap-seq and Infinium HumanMethylation450k BeadChips (HM450) are summarized for different beta-value thresholds: 0.2 (panel A), 0.3 (panel B) and 0.4 (panel C). In general, models converged moderately ($1.1 < \hat{R} <= 1.5$, grey) to very ($\hat{R} <= 1.1$, white) well. For a beta-value threshold of 0.2, there were slightly more samples with lack of convergence ($\hat{R} > 1.5$, black).

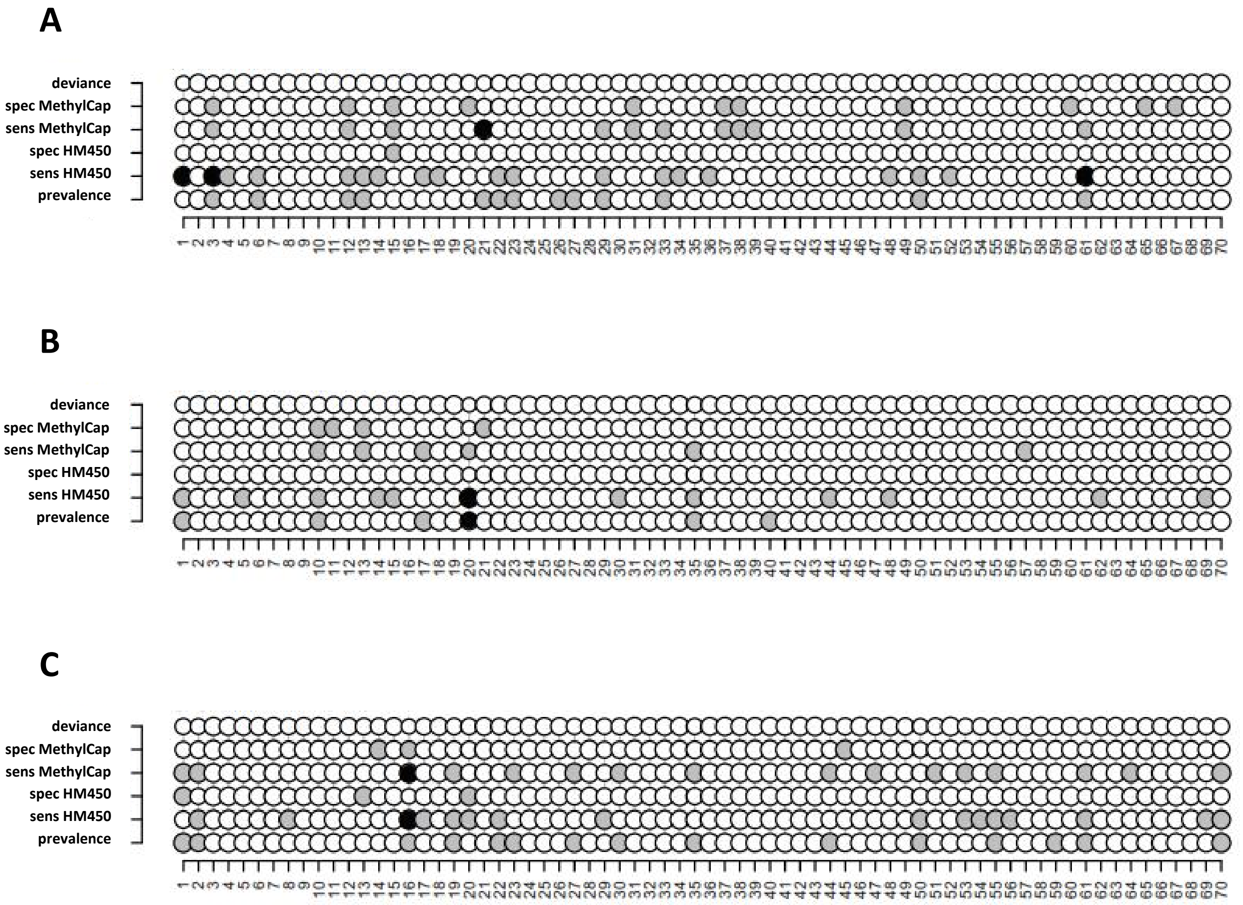

Figure S3 – Model convergence regarding deviance, prevalence, sensitivity and specificity estimation for assay type 2 loci. For each sample (X-axis), convergence results regarding deviance, specificity (spec), sensitivity (sens) and prevalence for both MethylCap-seq and Infinium HumanMethylation450k BeadChips (HM450) are summarized for different beta-value thresholds: 0.2 (panel A), 0.3 (panel B) and 0.4 (panel C). In general, models converged moderately (1.1 < $\hat{R}$ <= 1.5, grey) to very ($\hat{R}$ <= 1.1, white) well. For a beta-value threshold of 0.2, there were slightly more samples with lack of convergence ($\hat{R}$ > 1.5, black).

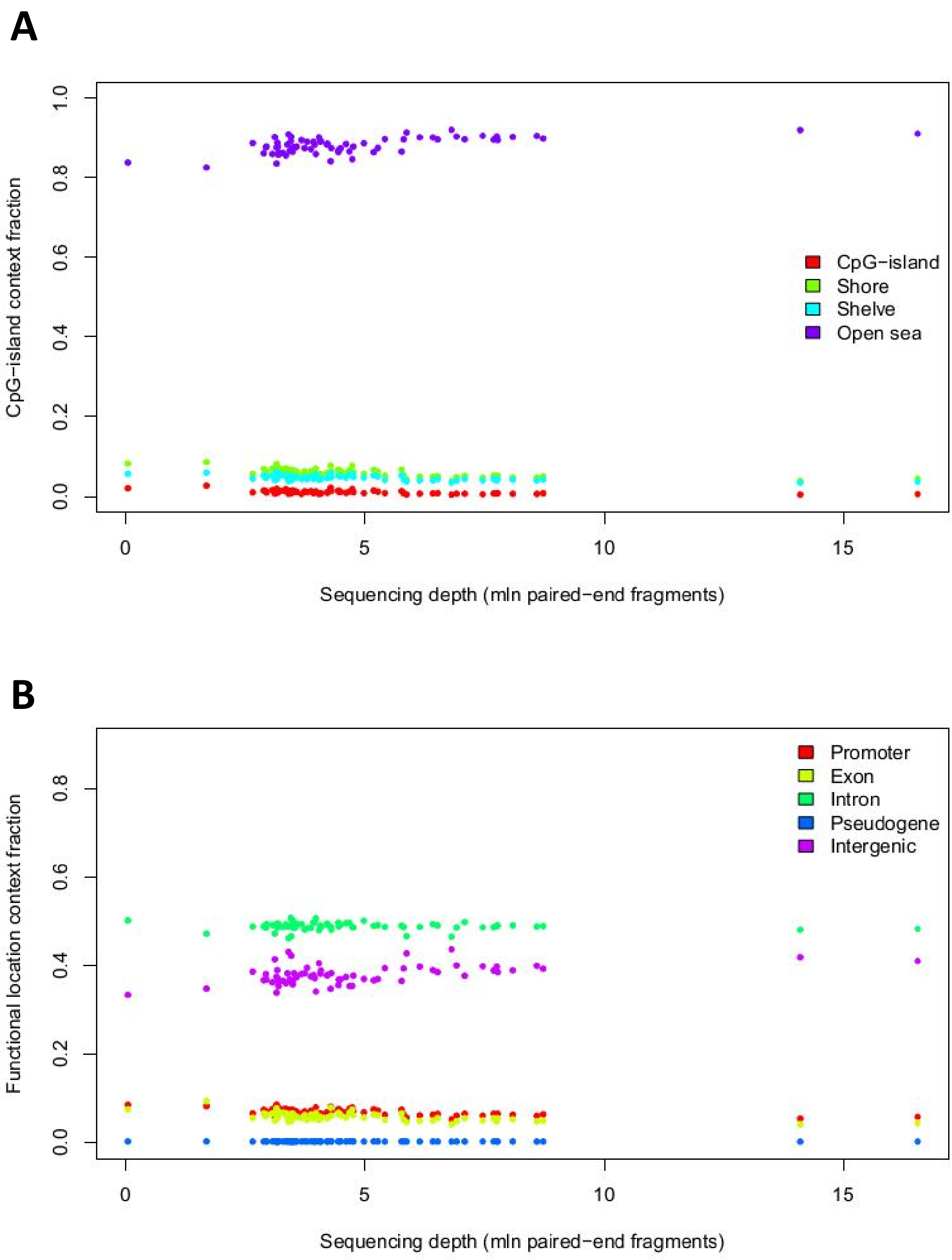

Figure S4 - CpG-island context (A) and functional location (B) context fractions as a function of MethylCap-seq sequencing depth. There is a significant increase of open sea (A) and intergenic (B) fractions, and a significant decrease of all other fractions - except introns - for higher sequencing depths (all $P < 0.005$, ANOVA).

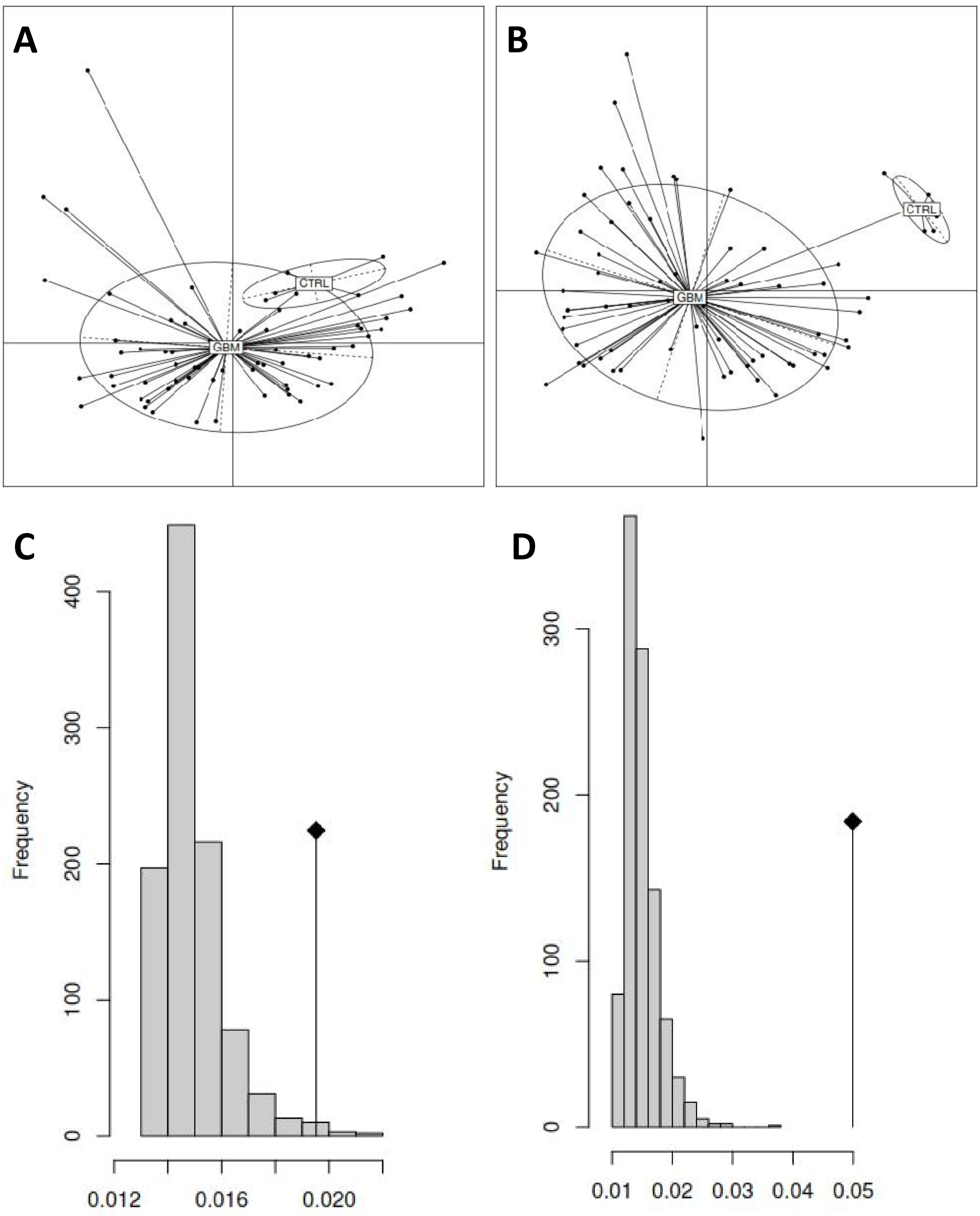

Figure S5 – Principal coordinate analysis (PCO) for MethylCap-seq (A, C) and HM450 data (B, D). PCO plots (A, B) as well as permutation based significance testing (C, D) indicate significant global differences between tumour (GBM) and control (CTRL) samples (MethylCap-seq: $P = 0.009$; HM450: $P < 0.001$). Histograms in panels C and D represent null distributions for 1000 permutations, observed differences are indicated by rhombuses and vertical lines.
